# Comparative study on the *in vitro *and *in vivo *properties of two bovine herpesvirus-5 reference strains

**DOI:** 10.1186/1751-0147-53-37

**Published:** 2011-06-08

**Authors:** María F Ladelfa, María P Del Médico Zajac, Fiorella Kotsias, Fernando Delgado, Benoît Muylkens, Julien Thiry, Etienne Thiry, Sonia A Romera

**Affiliations:** 1 Virology Institute, Veterinary and Agricultural Science Research Centre (CICVyA), National Institute of Agricultural Technology (INTA), N. Repeto y Los Reseros S/N, CC25 (B1712WAA), Castelar, Buenos Aires, Argentina; 2 Virology and Viral Diseases, Department of Infectious and Parasitic Diseases, Faculty of Veterinary Medicine, University of Liège, Boulevard de Colonster, 20, B43b, B-4000 Liège, Belgium; 3 Consejo Nacional de Investigaciones Científicas y Tecnológicas (CONICET), Rivadavia 1917 (C1033AAJ), Ciudad Autónoma de Buenos Aires, Argentina; 4 Pathobiology Institute, Veterinary and Agricultural Science Research Centre (CICVyA), National Institute of Agricultural Technology (INTA), N. Repeto y Los Reseros S/N, CC25 (B1712WAA), Castelar, Buenos Aires, Argentina

## Abstract

**Background:**

Bovine herpesvirus 5 (BoHV-5) is an alphaherpesvirus responsible for meningoencephalitis in young cattle and it is antigenically and genetically related to bovine herpesvirus 1. BoHV-5 outbreaks are sporadic and restricted in their geographical distribution, being mostly detected in the Southern hemisphere. The N569 and A663 strains are prototypes of the "a" and "b" subtypes of BoHV-5, however, scarce information about their *in vitro *and *in vivo *properties is currently available.

**Methods:**

For the *in vitro *comparison between BoHV-5 A663 and N569 strains, viral growth kinetics, lysis and infection plaque size assays were performed. Additionally, an experimental infection of cattle with BoHV-5 A663 and N569 strains was carried out. Viral excretion, development of neurological signs, presence of specific antibodies in serum and nasal swabs and presence of latent BoHV-5 DNA in trigeminal ganglion, were analyzed. Histopathological examination of samples belonging to inoculated animals was also performed.

**Results:**

The lytic capacity and the cell-to-cell spread was lower for the A663 strain compared to the N569 strain, however, the production of total infectious viral particles was similar between both strains. Concerning the *in vivo *properties, the A663 and N569 strains are able to induce similar degrees of pathogenicity in cattle.

**Conclusions:**

Our results show that the A663 strain used in this study is less adapted to *in vitro *replication in MDBK cells than the N569 strain and, although slight differences were observed, both strains are able to induce a similar degree of virulence in the natural host.

## Background

Bovine herpesvirus 5 (BoHV-5) is an alphaherpesvirus associated with meningoencephalitis in young cattle and it is antigenically and genetically related to bovine herpesvirus 1 (BoHV-1) [1-3]. BoHV-5 was former classified as a neuropathogenic variant of BoHV-1. In 1992, data based on restriction site mapping of viral DNA [4-6], cross-neutralization tests, and monoclonal antibody reactivity [7,8], allowed the International Committee on Taxonomy of Viruses to recognize BoHV-5 as a distinct virus from BoHV-1 [9].

Contrasting with the BoHV-1 worldwide distribution, BoHV-5 outbreaks are sporadic and restricted in their geographical distribution, being mostly detected in the Southern hemisphere. The reasons for this particular distribution are still undetermined. Sporadic cases of BoHV-5-associated encephalitis have been detected in Australia [10,11], North America [4,12] and Europe [13,14]. Outbreaks are most commonly reported in Brazil [15-17] and Argentina [18-20].

According to restriction endonuclease analysis, BoHV-5 strains are classified into three subtypes [8,21]. Type strains for subtypes "a", "b" and "non-a-non-b", are the Australian strain N569, the Argentinean strain A663 and Brazilian isolates, respectively. Despite the geographical proximity between Argentina and Brazil, most of the Brazilian isolates studied belong to the "a" subtype [21]. This discrepancy could be attributed to the small number of BoHV-5 isolates characterized to date in Argentina and Brazil. Besides, the isolates examined to date may not be actual representatives for the most prevalent viruses in Brazil, as well as A663 itself may not be a typical representative of most Argentinean BoHV-5 isolates currently circulating in this country. In line with this, recent analysis of Argentinean BoHV-5 isolates isolated from 1982 to 2007 revealed that the "a" subtype is the most prevalent in this country [22]. Further characterization of recently isolated BoHV-5 field strains from Argentina and Brazil will provide information about the subtypes currently circulating in these countries.

BoHV-5 infection induces different degrees of severity of neurological disease depending on both viral and host factors. Viral genes and their encoded proteins involved in the neurovirulence of alphaherpesviruses are classified in three groups: enzymes involved in nucleic acid metabolism, factors that modulate the immune response and viral glycoproteins (g). Regarding viral glycoproteins, a role in the anterograde transport of gI, gE and Us9 was suggested in the rabbit model [23,24]. Concerning host factors, the age and immunological status of the animals appear to be the most relevant ones [25].

Scarce *in vitro *studies to assess the growth properties of N569 and A663 strains have been performed. These studies allowed the identification of cell lines susceptible to BoHV-5 [10] and the establishment of growth curves [19]. Concerning *in vivo *properties, some experimental inoculations with BoHV-5 N569 and A663 strains have been carried out [8,26-28]. In these studies, neurological signs, such as bruxism, depression, anorexia and muscle trembling, were observed in calves infected with both BoHV-5 strains. Although BoHV-5-induced encephalitis is usually fatal in young animals, some experimentally infected calves develop subclinical infection [25,29,30] or moderate disease [27]. However, up to now, the *in vivo *studies of N569 and A663 strains were independently performed and carried out under different conditions. At this point, a direct comparison of the pathogenecity of these strains is difficult to make.

In this context, the aim of the present study is to compare the *in vitro *and *in vivo *properties of these two BoHV-5 reference strains belonging to different subtypes.

## Materials and methods

### Viruses and cell culture

The N569 BoHV-5 strain was isolated from a brain sample after several intracerebral inoculations of calves with brain tissue obtained from an outbreak of meningoencephalitis in calves reported in 1962 in Australia [10]. The A663 BoHV-5 strain was isolated from a case of non purulent encephalitis in an outbreak among calves in 1982 in Argentina [19].

N569 and A663 BoHV-5 strains were propagated in Madin Darby bovine kidney (MDBK) cells and viral stocks were produced after infection of MDBK at a low multiplicity of infection (MOI) as previously described [31].

#### Viral growth kinetic

To perform one-step kinetics, MDBK monolayers grown in 60 mm culture dishes were inoculated with BoHV-5 N569 or A663 at MOI 5. Cells were incubated at 4°C for 2 h in order to synchronize virus adsorption. Then, cells were incubated at 37°C for 2 h and treated with low pH solution (40 mM citric acid, 10 mM KCl, 135 mM NaCl) for 2 min to inactivate the remaining extracellular virus. Monolayers were rinsed with PBS, E-MEM 2% FCS was added and the dishes were incubated at 37°C. At 0; 3; 6; 12; 15; 18 and 24 h post infection (hpi) the extracellular and total fractions were obtained. These fractions were titrated twice on MDBK monolayers in duplicate and viral titres were calculated by the Reed and Muench method [31]. Mean of viral titres were compared by Mann-Whitney no-parametric test, *P*< 0.05 (Software MedCalc^® ^statistical software version 11.1.1.0).

### Lysis and infection plaque size assays

MDBK monolayers grown in 12 wells culture plates were inoculated with BoHV-5 N569 or A663 at tenfold serial dilutions. After 2 h of incubation at 37°C, the inoculum was removed and 2 ml of carboxy methyl cellulose (CMC) 1.5% FCS per well were added. Plates were incubated at 37°C for 72 h.

For the lysis plaque size assay, cells were fixed with 10% formaldehyde for 10 min at room temperature, stained with violet crystal (1% violet crystal, 10% ethanol in PBS) for 20 min and washed in PBS. For each virus, 60 isolated and randomly selected lysis plaques were observed with an optical microscope and photographed.

For the infection plaque size assay, cells were fixed with 4% paraformaldehyde (PFA) for 10 min at room temperature. After washing in PBS, cells were incubated for 1 h at 37°C with an anti IBR-FIT-C conjugated antibody (VMRD, Inc. Pullman, U.S.A) diluted in PBS and then cells were washed twice in PBS. For each virus, 30 isolated and randomly selected infectious foci were observed with a fluorescent microscope and photographed.

The photographs were analyzed with ImageJ software in order to calculate lysis and infection plaques surfaces.

### Animal experimental design

Ten 3-month-old calves were housed in an experimental unit two weeks before infection. Upon arrival and before experimental infection, their naïve status for BoHV-1 and 5 exposures were verified by ELISA, seroneutralization and lack of viral isolation from nasal swabs. Calves were separated in 2 groups of four animals each and one group of two animals (control group, uninfected animals). The groups were strictly isolated from each other during the course of the study in order to avoid viral spread and contamination. Animal care and experimental procedures were performed in accordance with the requirements of the National Institute of Agricultural Technology Ethics Committee (INTA, Argentina).

Two groups were infected by aerosolization with 3 ml of E-MEM containing a total dose of 10^6.5 ^TCID_50_/ml (1.5 ml in each nostril) of N569 or A663 BoHV-5 strains. Control group received 3 ml of E-MEM. Clinical observation of respiratory and nervous signs, and rectal temperature were recorded in an individual sheet for each animal. Between days 35-37 post infection (pi) animals were sedated with acepromazine (Asedan, Holliday Laboratories, San Isidro, Argentina) by intramuscular route and then euthanized by a barbiturate overdose (Euthanyle, Brouwer Laboratories, Ciudad Autónoma de Buenos Aires, Argentina). One animal from the BoHV-5 A663 group developed severe neurological signs and was euthanized at day 15 pi. For all animals, necropsy was performed immediately after euthanasia. Transverse sections from the cervical spinal cord, olfactory cortex, frontal, parietal and occipital cortex, thalamus, mesencephalon, cerebellum, trigeminal ganglia and respiratory tract tissues were collected. To perform histological examination, the samples were fixed in 10% neutral buffered formalin, prepared by routine methods for histology, paraffin embedded, sectioned at 4 μm and stained with hematoxylin and eosin. The histopathological CNS alterations were interpreted as follows: +++: severe; ++: moderate; +: slight; -: none.

### Sampling procedure, serological and virological analysis

Blood samples were taken on the day of inoculation and then weekly by jugular venipuncture to monitor antibody levels. Sera obtained after centrifugation were stored at -20°C until analyzed. Serum antibodies against BoHV-1 and -5 were detected by ELISA and seroneutralization [31].

Nasal secretions were collected inserting tampons in the ventral meatus of the nasal cavity for 5 min (to assure the absorption of nasal fluids potentially containing viral particles into the tampon) and thereafter immediately dipped in 5 ml E-MEM containing 5000 IU penicillin/ml, 2500 μg streptomycin/ml and 10 μg amphotericin B/ml [31]. Tampons were centrifuged and samples were stored at -80°C until used. Nasal samples were taken daily from day 0 to 18 pi and then two times per week until the end of the study. Nasal swabs were inoculated immediately after collection onto MDBK cell monolayers: 0.1 ml of nasal fluids was inoculated onto 96 well microtitre plates and tenfold serial dilutions were tested in four wells. Monolayers were inspected until cytopathic effect appeared and virus titres were calculated by the Reed and Muench method [32]. IgA antibodies in nasal secretions were determined by ELISA as previously described [27].

### Detection of latent BoHV-5 DNA in trigeminal ganglion

Pieces of approximately 100 mg of trigeminal ganglion were digested and total DNA extracted using the QIAamp DNA Mini kit (Qiagen, Spain). In order to examine the presence of viral DNA, a polymerase chain reaction (PCR) that amplifies BoHV-5 gD gene was performed [33]. PCR was also performed with a set of primers generating a 250 base pair fragment from the bovine *glyceraldehyde-3-phosphate dehydrogenase *gene (GAPDH), selected as bovine housekeeping gene. The primers were designated as GAPDH-Fow (5'-GCA TCG TGG AGG GAC TTA TGA) and GAPDH-Rev (5'-GGG CCA TCC ACA GTC TTC TG).

## Results

### *In vitro *characterization

With the aim of performing an *in vitro *comparison between N569 and A663 BoHV-5 strains, their growth properties, lysis and infection plaque size were assessed.

**Figure 1:**
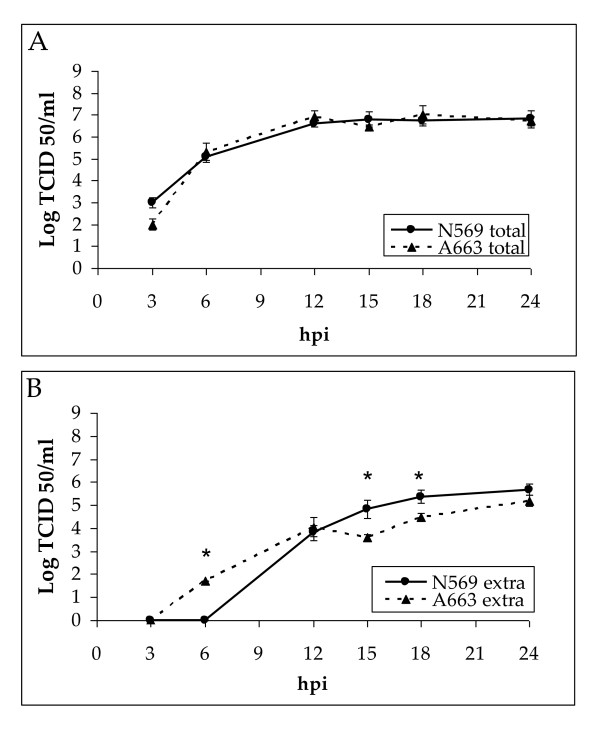
**One-step growth kinetics of bovine herpesvirus-5 N569 and A663 strains (MOI 5)**. **A**: total fraction viral titres. **B**: extracellular fraction viral titres. Viral titres are expresed as Log_10 _TCID_50_/ml. * N569 and A663 are significantly different (Mann-Whitney, *P *< 0.05).

The one-step growth kinetics curves profiles for the N569 and A663 strains of BoHV-5 were established. Total fractions curves showed similar profiles and the amount of infectious virus produced was also similar between both strains at each time assayed (Figure 1A). Significant differences between A663 and N569 viral titres in their total fractions at each time assayed were not found. Maximum titres were obtained at 24 (10^6.8 ^TCID_50_/ml) and 18 (10^7.0 ^TCID_50_/ml) hpi for N569 and A663, respectively.

As shown in Figure 1B, the release of infective viral particles to the extracellular media was first detected for A663 strain (at 6 hpi). However, the viral titre reached by this strain from 15 to 24 hpi was slightly lower than the titre reached by N569 strain. Although significant differences between N569 and A663 viral titres were observed at 15 and 18 hpi, at the end of the assay (24 hpi) both strains showed similar viral titres in their extracellular fractions.

In order to study the lytic capacity of both BoHV-5 strains, the lysis plaque size assay was performed. This parameter contributes to the *in vitro *characterization of viruses because it represents a direct measure of viral lytic potential and an indirect measure of viral cell-to-cell spread.

**Figure 2:**
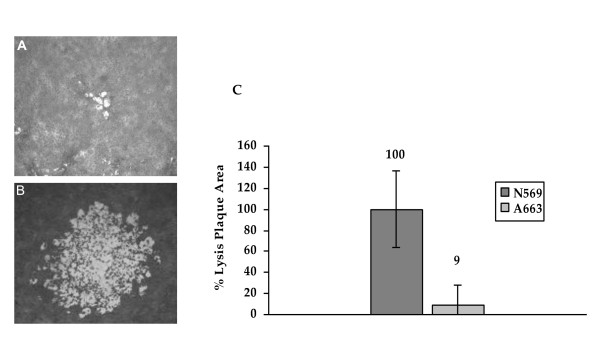
**Lysis plaques generated by bovine herpesvirus-5 A663 (**A**) and N569 (**B**) strains**. Magnification Obj. 40×. **C**: Size rate between both strains. The N569 mean area was established as 100%. Error bars represent standard deviation. N569 and A663 are significantly different (Mann-Whitney, *P *< 0.01).

The comparison of the lysis plaque size after the inoculation of MDBK monolayers with the N569 and A663 strains, shown a sharp difference, being the plaques generated by the A663 strain 90% smaller than those generated by the N569 strain (Figure 2). This result is in agreement with the lower viral titres observed in the extracellular fraction of the one-step growth kinetics of the A663 strain in comparison with those of the N569 strain.

To further analyze the cell-to-cell spread of the N569 and A663 strains, the infection plaque size assay was performed. This assay takes into consideration the cells lysed by the virus and also the infected cells expressing viral antigens on their surface, being therefore an appropriate tool for studying cell-to-cell dissemination.

**Figure 3:**
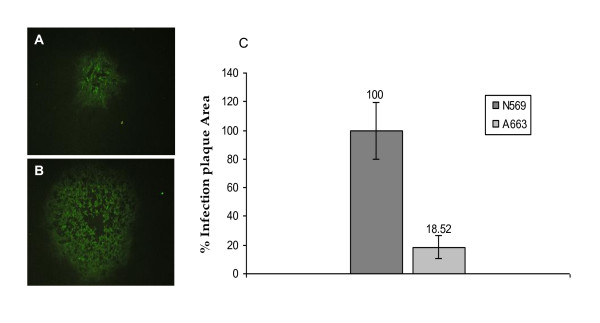
**Infection plaques generated by bovine herpesvirus-5 A663 (**A**) and N569 (**B**) strains**. Magnification Obj. 40×. **C**: Size rate between both strains. The N569 mean area was established as 100%. Error bars represent standard deviation. N569 and A663 are significantly different (Mann-Whitney, *P *< 0.01).

The comparison between the infection plaques generated by N569 and A663 strains showed a sharp difference, being the plaques generated by the A663 strain 80% smaller than those generated by the N569 strain (Figure 3).

### *In vivo *characterization

In order to perform an *in vivo *comparative study of the reference BoHV-5 strains, two groups of 4 calves were infected with N569 (subtype "a") or A663 (subtype "b") strains. A mock infected group of two animals was included (control group).

After intranasal infection, both groups showed a similar time period of viral excretion (14 days). Three out of four animals infected with N569 strain presented viral particles in nasal swabs, one of them showing an intermittent profile of viral excretion (Table 1). All the animals infected with A663 strain shed virus, three of them excreted during 5-13 days and the remaining one showed intermittent excretion (Table 1). The profiles of viral shedding were similar between both groups in the studied period. Control animals did not excrete virus during the studied period.

**Table 1:** Viral titres shedding after experimental infection of calves (n = 8) with N569 and A663 strains of bovine herpesvirus (BoHV)-5.

Grupo	**Animal no**.	Days post infection													
		
		2	3	4	5	6	7	8	9	10	11	12	13	14	15
**BoHV-5 N569**	628	Nd	Nd	Nd	Nd	Nd	2	Nd	**5**	Nd	3.5	3.5	3.5	3.3	2.5
	
	191	Nd	Nd	Nd	Nd	Nd	Nd	Nd	Nd	Nd	Nd	Nd	Nd	Nd	Nd
	
	199	1.6	**4.3**	Nd	Nd	3.3	Nd	Nd	Nd	Nd	Nd	Nd	Nd	Nd	Nd
	
	636	2.6	Nd	5	5.3	Nd	4.3	**6**	3	4.5	Nd	3	Nd	Nd	Nd

**BoHV-5 A663**	254	3	2.3	5	4.5	4.5	**5**	3.5	Nd	3.5	1.6	3.3	Nd	Nd	2
	
	619	3.6	5.3	**5.5**	**5.5**	5	Nd	Nd	Nd	Nd	Nd	Nd	Nd	Nd	Nd
	
	192	3.5	4	4.6	5.3	6	**6.5**	6.3	5	5.3	3.5	3.5	2	1.6	Nd
	
	185	Nd	Nd	**2.5**	Nd	Nd	Nd	Nd	Nd	Nd	2.3	2	Nd	Nd	Nd

Nd: Non detected

Titres are expressed as log TCID50/ml of nasal secretions. For each animal, the peak titer of viral excretion is highlighted in bold.

Clinical signs associated with BoHV-5 infection were observed in one A663 infected calf. This animal showed breathing difficulties and a slight ptyalism at day 11 pi. Nervous signs started day 12 pi and were characterized by bruxism, ptyalism, muscular tremor, circular movements, ataxia and seizure-like episodes. At day 15 pi this animal was euthanized according to ethical considerations. None of the animals infected with BoHV-5 N569 strain developed nervous signs during the studied period. At days 35-36 pi the surviving animals were sacrificed and necropsy was performed.

Histopathological examination of samples belonging to BoHV-5 N569 and A663 groups revealed the presence of severe lesions in the CNS associated with BoHV-5 infection in two out of four animals in each group. These animals presented non suppurative meningoencephalitis in the olfactory cortex, frontal, parietal and occipital cortex, thalamus, mesencephalon and cerebellum. Focal gliosis was also found. Mild infiltration by macrophages in the mesencephalon, thalamus, cerebellum and spinal cord were observed. The other two animals from BoHV-5 A663 strain group showed moderate or slight lesions in all sections examined meanwhile the other two animals infected with N569 did not show significant alterations in the CNS (Table 2).

**Table 2:** Data obtained from calves (n = 8) experimentally inoculated with A663 and N569 strains of bovine herpesvirus (BoHV) 5.

Group	**Animal no**.	Viral excretion* (peak titre)	Severity of histopathological changes (mean)**	DNA in TG	SN Abs titre(log)***	Nasal IgA
BoHV-5 N569	636	2-12 (6)	+++	+	1.5	+
	
	628	7-15 (5)	++	+	1.05	-
	
	199	Intermittent (4.3)	-	-	1.5	+
	
	191	-	-	-	-	-

BoHV-5 A663	192	2-12 (6.5)	+++	+	0.9****	-
	
	619	2-6 (5.5)	+++	+	0.9	+
	
	185	Intermittent (2.3)	+	+	0.9	+
	
	254	2-12 (6.3)	+	+	0.9	+

*Viral excretion is expressed as log TCID_50_/ml. **Score based on the presence of the following CNS histopathological alterations: vascular alteration, inflammation, focal gliosis, infiltration by macrophages and malacia. References: +++: severe; ++: moderate; +: slight; -:negative. ***Serum neutralizing (SN) antibodies (Abs) at day 34 post infection (pi). ****Animal 192 was euthanized 15 days pi, the titre of SN Abs corresponds to this date.

Next, the establishment of latency of both BoHV-5 strains was examined. In order to test the presence of latent BoHV-5 DNA, PCR assays were performed using samples from trigeminal ganglia dissected after euthanasia. Viral DNA was detected in all animals from A663 group and in two animals from N569 group (Table 2). In addition, trigeminal ganglia were assessed for induction of cytopathic effect in susceptible cells. Since no cytopathic effect was developed in the cultures, we concluded that the positive PCR results were due to the presence of latent viral genomes.

With a view to evaluating seroconversion specifically raised against BoHV-5 after the experimental infection, serum antibodies and mucosal IgA were analysed. Serum antibodies either measured by ELISA or by SN assay were detected in seven out of eight BoHV-5 infected animals. Moreover, we detected nasal IgA in five out of eight infected animals (Table 2).

## Discussion

The real prevalence of BoHV-5 is unknown because outbreaks are sporadic and routine serologic tests do not discriminate between antibodies against BoHV-5 and BoHV-1. Due to that, BoHV-5 sanitary and economic impact may be underestimated. This, in addition to the scarce information available on BoHV-5 biology, has prompted the present study.

The *in vitro *characterization performed here suggests that the A663 strain (BoHV-5 subtype "b") possesses diminished capacities concerning cell lysis and cell-to-cell spread in comparison with the N569 strain (BoHV-5 subtype "a"). In addition, the *in vivo *properties of A663 and N569 were analyzed and, as a result, we observed that both BoHV-5 strains induced a similar degree of virulence in cattle.

Regarding the *in vitro *characterization, the results obtained from the one-step growth kinetic showed that the production of total infectious viral particles was similar between both BoHV-5 strains. However, the release of infectious viral particles to the extracellular media was lower for the A663 strain. The one-step growth kinetic was performed at high MOI, thus, all cells were infected and cell-to-cell spread was not being evaluated. Hence, the lower release of A663 infectious viral particles to the extracellular media suggested a lower lytic capacity for this strain. This suggestion was directly confirmed by the lysis plaque size assay, which showed that lysis plaques generated by A663 were 90% smaller than those generated by N569. Indeed, this dramatic difference led us to suggest that the A663 strain has a less effective cell-to-cell spread in comparison with N569. In this respect, multiple-steps growth kinetic (performed at low MOI) showed the viral titres in both extracellular and total fractions were larger for N569 than for A663, supporting the contribution of cell-to-cell spread to the lysis plaque area reduction observed for the A663 strain (data not shown). To further assess this, the infection plaque size assay was performed and showed that A663 plaques were 80% smaller than those generated by N569 strain. In this context, we concluded that both lytic capacity and cell-to-cell spread are diminished in the A663 strain in comparison with N569. The fact that A663 and N569 reach the same viral titres in their total fractions in the one-step growth kinetics (from 3 to 24 hpi) indicates that viral particles are equally produced by both strains, however, A663 viral particles could be retained for longer times inside the cell due to their difficulties in cell-to-cell spread and cell lysis.

Some aspects of the *in vitro *viral replication discussed here could have some relevance *in vivo*, for example: i) *in vitro *total production of infectious virus could be related with *in vivo *levels of viral excretion, or ii) *in vitro *cell-to-cell spread could have consequences on viral dissemination and infection of different animal tissues, or iii) the lytic capacity could be associated with the level of tissues damage and therefore with the clinical signs observed after viral infection. All these correlations could help understanding the fact that "b" subtype of BoHV-5 has only been reported in Argentina [21,34] and that it seems to have disappeared since it has not been found circulating during the last years [22]. As suggested by our *in vitro *results, A663 strain could have diminished capacities to be maintained in the cattle population, in contrast with "a" subtypes of BoHV-5, which circulation among Argentinean cattle herds have been recently reported for first time [22].

The differences observed in the lysis and the infection plaque sizes between both BoHV-5 strains are larger than expected and have not been reported for wild type strains of the same alphaherpesvirus previously. Despite the fact that the viruses used to perform these assays had the same number of cell culture passages (n = 8), the previous passages to this experience on the original viral stocks were unknown. In our work, passage 8 of N569 strain was obtained after successive passages in cell culture from virus excreted by an animal infected with a N569 virus of unknown, and thus high, number of passages. For this strain, no difference in lysis plaque size was observed between passage 8 and the virus of high number of passages used to infect the bovine (data not shown). Concerning A663 strain, passage 8 of A663 strain was also obtained after successive passages in cell culture from virus secreted by an animal infected with this strain, but in this case, the strain used to infect the animal had a low number of passages. Contrary to N569, a sharp difference between A663 passage 8 and A663 of high number of passages was observed in lysis plaque size, being those caused by A663 passage 8 much smaller. This indicates that plaque size increased with the number of passages in cell culture of the non-adapted to cell culture A663 strain (data not shown). Taking all this into consideration, we are not able to exclude differences between both strains strictly due to cell culture adaptation. Additionally, the genetic background of N569 and A663 strains is unknown and we are not able to rule out the possibility of genomic differences having an impact on the *in vitro *behavior of both BoHV-5 strains.

Concerning the *in vivo *characterization, in spite of the *in vitro *differences discussed above, we observed that both BoHV-5 strains induced a similar degree of virulence in cattle. Almost all animals infected with the N569 or A663 strains showed moderate to high levels of viral excretion confirming the virulence of the viruses used. Individual susceptibility or technical problems during the viral inoculation could account for the lack of infection of the animal 191 from the N569 group.

Only one animal infected with A663 strain developed clinical signs associated with BoHV-5 infection. This difficulty in reproducing clinical encephalitis in BoHV-5 infected animals was also faced by others [29,35]. Indeed, BoHV-5 infection could induce different degrees of disease. In this respect, Meyer *et al. *[26], infected 3-month-old calves with N569 BoHV-5 strain and the animals developed severe neurological signs. Additionally, 6 to 8-month-old animals experimentally infected with the A663 strain developed a moderate BoHV-5-induced neurological disease [27]. In other studies, experimental infections in 4 to 6-month-old calves with BoHV-5 TX74 and EC-1 field isolates [25,29] and Brazilian isolate SV-507 [30], have been performed without subsequent development of neurological symptoms, although histopathological changes in the CNS were observed. In accordance with these studies, we did not observe neurological signs in most of the infected animals but histopathological examination of CNS samples evidenced the development of BoHV-5-associated encephalitis. Because the age of the animals and the viral dose used in this study were similar to those of previous studies in which clinical signs were observed [26,27], the lack of neurological signs reported here could be due to the individual immunological status of cattle.

In addition, in agreement with results presented elsewhere [36], latency was detected in all the animals infected with A663 strain and two out of four animals infected with N569. The negative result obtained from trigeminal ganglion belonging to animal 199 could be due to technical problems with conservation and/or processing of the sample. In the case of animal 191, as discussed above, the reason could have been the lack of infection, as all the other parameters assayed were also negative for this animal.

At this point, we could suggest that although the development of neurological signs is not always achieved under experimental infection with BoHV-5, presence of lesions in the CNS and establishment of latency are more frequently observed.

Finally, we showed that four out of four and three out of four animals seroconverted after infection with A663 and N569 BoHV-5 strains, respectively. In addition, three out of four and two out of four animals infected with A663 and N569, respectively, presented IgA mucosal antibodies. This constitutes the first report of IgA in nasal secretions of animals infected with BoHV-5 and is in agreement with previous reports showing induction of nasal IgA in calves after infection with different BoHV-1 strains [37]. Concerning animals 628 and 192, the lack of IgA detection could be due to the sensitivity of the ELISA test available. In the case of animal 191, as discussed above, the reason could have been the lack of infection.

## Conclusions

The *in vitro *and *in vivo *properties of two strains of BoHV-5 belonging to different subtypes were studied. Our results show that the A663 strain used in this study is less adapted to *in vitro *replication in MDBK cells than the N569 strain and, although slight differences were observed, both strains are able to induce a similar degree of virulence in the natural host. These results also highlight the importance of considering viral *in vitro *adaptation previous to study *in vivo *properties and draw general conclusions about the biology of BoHV-5. To study the plausible differences in virulence associated to BoHV-5 subtypes, we think it would be suitable to use BoHV-5 field isolates without previous adaptation to cell culture instead of viral strains with unknown history. Taking all this into consideration, further research should be done in order to better understand the biology of BoHV-5 and the relevance of its subtypes.

## Competing interests

The authors declare that they have no competing interests.

## Authors' contributions

MFL and SAR designed the experiments, analysed the data and drafted the manuscript. MFL, MPDMZ, FK and SAR, performed the experiments. BM, JT and ET participated in the *in vitro *characterization studies and interpretation of data. FD carried out the histopathological analysis of the samples. All authors read and approved the final manuscript.
